# Beyond sentinel node algorithm. Toward a more tailored surgery for cervical cancer patients

**DOI:** 10.1002/cam4.722

**Published:** 2016-05-27

**Authors:** Anna Fagotti, Luigi Pedone Anchora, Carmine Conte, Vito Chiantera, Enrico Vizza, Lucia Tortorella, Daniela Surico, Pierandrea De Iaco, Giacomo Corrado, Francesco Fanfani, Valerio Gallotta, Giovanni Scambia

**Affiliations:** ^1^ Division of Minimally Invasive GynaecologySt. Maria HospitalUniversity of PerugiaTerniItaly; ^2^ Gynecologic Oncology UnitDepartment of Obstetrics and GynaecologyCatholic University of the Sacred HeartRomeItaly; ^3^ Division of Gynecologic OncologyDepartment of OncologyFoundation John Paul IICatholic University of the Sacred HeartCampobassoItaly; ^4^ Gynecology Oncology UnitDepartment of Oncological Surgery“Regina Elena” National Cancer InstituteRomeItaly; ^5^ Department of Obstetrics and GynecologyUniversity of Eastern PiedmontNovaraItaly; ^6^ Division of Gynecologic OncologyDepartment of Obstetrics and GynecologyS. Orsola HospitalUniversity of BolognaBolognaItaly

**Keywords:** Cervical cancer, early stage, lymphadenectomy, sentinel lymph node

## Abstract

Nowadays cervical cancer is frequently diagnosed at early stage. For these patients lymph node metastasis (LNM) is considered the most important prognostic factor. During the last decade many efforts have been made to reduce rate of complications associated with lymphadenectomy (LND). A great interest has arisen in sentinel lymph node (SLN) biopsy as a technique able to decrease number of LND performed and, at the same time, to assess lymph nodal status. High diagnostic performances have been reached thanks to SLN surgical algorithm. However, despite the efforts, about 25% of these patients undergo at least unilateral LND to meet NCCN recommendations. Data of women with International Federation of Gynecology and Obstetrics stage IA1‐IB1/IIA1 cervical carcinoma were retrospectively collected by six Italian institutions. All patients underwent complete preoperative staging workup and were primarily treated by radical hysterectomy and pelvic bilateral LND. A total of 368 patients with early‐stage cervical cancer were identified. Among them 333 (90.5%) showed no suspicious enlarged nodes at the preoperative magnetic resonance imaging (MRI). In this subset, tumor diameter ≥20 mm was the only independent predictor of LN status (*P* = 0.003). None of the 106 patients with negative MRI nodal assessment, with squamous and adenosquamous histotype and a tumor diameter less than 2 cm had LNM. Based on these results we propose a new modified SLN surgical algorithm that could safely reduce LND performed in patients with very low‐risk early‐stage cervical cancer.

## Introduction

Thanks to improvements in screening programs, cervical cancer is more frequently diagnosed in early stage of disease 1. Although the International Federation of Gynecology and Obstetrics (FIGO) staging system does not include lymph node (LN) status 2, presence of lymph node metastasis (LNM) is considered the most important prognostic factor 3, 4, 5 in cervical cancer patients.

Traditionally, to obtain histological diagnosis of nodal spread, all pelvic LNs are removed. However, pelvic/aortic lymphadenectomy (LND) is associated with short‐ and long‐term morbidities, such as prolonged duration of surgery, increased blood loss, infection, nerve and vascular injury, venous thromboembolism, lymphocyst formation, and lower extremity lymphedema 6, 7, 8, 9. In an effort to decrease complications associated with “unnecessary” LND and, at the same time, to assess LN status, sentinel lymph node (SLN) biopsy technique has been developed and investigated by multiple groups with encouraging data in terms of sensitivity and negative predictive value (NPV) 10, 11, 12. The idea of using sentinel node biopsy is based on the fact that status of regional LNs could be accurately represented by the status of the SLN. However, regardless of the technique used, detection rate of SLN ranges from 92% to 97% of overall mapping to 70–79% of complete, bilateral, mapping 13, 14. In order to ensure the detection of all LNM, an algorithm that recommends side‐specific nodal dissection in case of failed SLN mapping and removal of all suspicious enlarged nodes was developed and introduced in international guidelines 15, 16. In this algorithm, at least 25% of patients with early‐stage cervical cancer still undergo pelvic LND, with no data reported on their metastatic rate until now.

The objective of this study was to identify a subgroup of patients in whom it is possible to avoid unnecessary systematic LND, either in case of failed SLN mapping, or regardless of SLN mapping.

## Patients and Methods

This is a retrospective, multicenter study. Data for the current analysis were provided from six different Italian institutions (Catholic University of the Sacred Heart–Rome; Foundation for Research and Care “Giovanni Paolo II”–Campobasso; “Regina Elena” National Cancer Institute–Rome; “Sant'Orsola‐Malpighi” Hospital–Bologna; St Maria Hospital–Terni; University East Piedmont “A. Avogadro”–Novara). Institutional review board approval was obtained from each Center. All women included in the study had a proved diagnosis FIGO stage IA1‐IB1/IIA1 cervical carcinoma with negative LNs assessment at preoperative imaging by magnetic resonance imaging (MRI). Patients undergoing neo‐adjuvant therapy before surgery were excluded.

All cases were primarily treated by radical hysterectomy and systematic pelvic bilateral LND 17. No patient received SLN biopsy during the study period. Para‐aortic LND was performed at attending discretion and, anyhow, if pelvic LNs were found to be positive at frozen section analysis. Dedicated pathologists at each center performed histological diagnosis. Adjuvant treatment was suggested according to NCCN guidelines 18. Imaging assessment by FDG‐PET/CT, CT scan, or MRI was done at completion of adjuvant treatment. Women were followed up and monitored by clinical examination every 3 months for 2 years, every 6 months for 3 years, then annually until death. During follow up, the need for imaging was triggered by clinical symptoms. Disease progression was defined according to NCCN criteria 18.

Patient demographics, medical, surgical, pathological, and follow‐up data were retrospectively collected independently by each institution and subsequently collected together in an electronic database.

All statistical calculations were performed by SPSS software, version 17.0 (SPSS, Chicago, IL).

## Results

From February 2000 to March 2015, 368 patients with early‐stage cervical cancer were identified. Among them, 333 met inclusion criteria and were included in the analysis (Table 1). Median age was 46 years (range 25–76) and median body mass index was 23.5 kg/m^2^ (range 17–51). Majority of patients were diagnosed as having squamous carcinoma at FIGO stage IB1 (289/333, 86.8%). Type of radical hysterectomy was classified according to Querleu and Morrow 17 and distributed as follows: 17 (5.1%) type A, 161 (48.3%) type B, and 155 (46.6%) type C. Surgery was performed by Minimally invasive surgery (MIS) (either laparoscopy or robotics) in 143 cases (42.9%). All patients received pelvic LND, and a median of 28 (range 14–80) pelvic LNs was registered. Thirty‐two women (9.6%) had positive pelvic nodes at definitive histology, with a MRI NPV of 90%. Thirty‐seven patients underwent aortic LND, with a median of 14 (range 2–37) LNs removed. Among them, four (10.8%) patients were found as having aortic LNM. One hundred and fourteen patients (34.4%) underwent adjuvant therapy: 52 (45.4%) received chemo‐radiation, 56 (49.3%) radiotherapy alone, and six (5.2%) chemotherapy alone. The median follow up was 37 months (range 10–158). Five years progression‐free survival (PFS) and overall survival (OS) rates were 92.5% and 95.6%, respectively, in the entire population.

**Table 1:** Demographic and clinicopathologic characteristics of all patients

Characteristics	Patients, *n* (%)
All	333
Median age (years) (range)	46 (25–76)
Median BMI (kg/m^2^) (range)	24 (17–51)
Median tumor diameter (mm) (range)	17 (1–40)
FIGO stage	
IA 1/2	41 (12.3)
IB 1	289 (86.8)
IIA 1	3 (0.9)
Histology	
Squamous carcinoma	229 (68.8)
Adenocarcinoma	81 (24.3)
Adenosquamous	15 (4.5)
Clear cell	4 (1.2)
Other	4 (1.2)
Grading	
1	29 (8.8)
2	152 (45.6)
3	152 (45.6)
Median no. pelvic LNs removed (range)	28 (14–80)
No. of cases with positive pelvic LNs	32 (9.6)
No. of cases submitted to aortic LND	37 (11.1)
Median no. aortic LNs removed (range)	14 (2–37)
No. of cases with positive aortic LNs	4 (10.8)

BMI, body mass index; FIGO, International Federation of Gynecology and Obstetrics; LNs, lymph nodes; LND, lymphadenectomy.

Table 2 shows the distribution of LN metastases according to patient and tumor characteristics. At multivariate analysis tumor diameter ≥20 mm was the only independent predictor of LN status in this patient population. Interestingly, among patients with tumor volume less than 2 cm, only one (0.7%) woman had LNM. She was a 49‐year‐old woman with a G2 adenocarcinoma and a tumor diameter of 17 mm having metastasis in two pelvic LNs.

**Table 2:** Clinicopathologic factors related to LNM.^a^

			Analysis	
Factor	LNM		Univariate	Multivariate
	No	Yes	*P*‐value^b^	*P*‐value^c^
Age				
<46	141	16		
≥46	160	16	0.438	–
FIGO stage				
IA 1/2	41	0		
IB 1	258	31		
IIA 1	2	1	**0.035**	0.153
Histology				
Squamous carcinoma	209	20		
Adenocarcinoma	74	7		
Adenosquamous	11	4		
Clear cell	3	1		
Other	4	0	0.145	–
Grading				
1	29	0		
2	140	12		
3	132	20	0.054	–
Tumor diameter (mm)				
<20	176	1		
≥20	125	31	**<0.001**	**0.003**

LNM, lymph node metastasis; FIGO, International Federation of Gynecology and Obstetrics.

a Only variables with a *P* < 0.05 at univariate analysis were included in multivariate analysis. Variables with a *P* < 0.05 were highlighted in bold

b Chi‐squared test or Fisher exact test.

c Logistic regression analysis.

Therefore, the combination of histopathological characteristics (squamous and adenosquamous histotype) and tumor diameter less than 2 cm along with preoperative MRI nodal assessment allowed to reach a NPV of 100%, with respect to the risk of unexpected LNM. This group of women was named very low‐risk group (VLRG), as no LNM was found among 106 patients meeting inclusion criteria. In the VLRG, 14 women (13.2%) received adjuvant radiotherapy, based on histopathological risk factors 18. With a median follow up of 40 months (16–158), seven (6.6%) recurrences have been registered: five central pelvic (one with LNs) and two distant metastases. None had exclusive lymphnodal recurrence. Among those patients who recurred in the VLRG, two women had multiple risk factors at definitive histological diagnosis (G3, deep stromal invasion, and/or lymphovascular space invasion), requiring adjuvant treatment. However, they did not receive radiotherapy due to the presence of large infected lymphocele and sepsis in one case and refusal in the other one. No risk factors were observed in the remaining five cases. The rate of relapse in the no‐VLRG was 8.8% (20 of 227) and no statistically significant difference was registered between the groups (VLRG vs. no‐VLRG recurrence rate *P* = 0.32).

**Figure 1:**
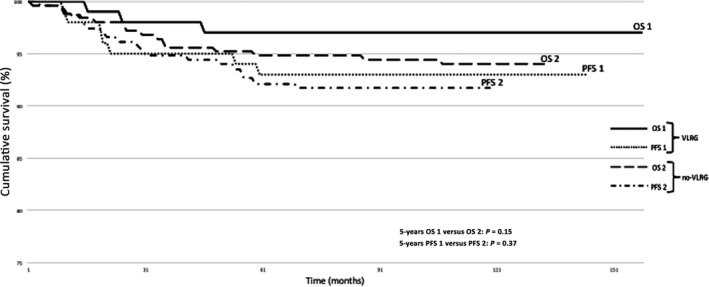
Progression‐free survival (PFS) and overall survival (OS) in very low‐risk group (VLRG) and no‐VLRG patient population.

Figure 1 shows PFS and OS in the VLRG and no‐VLRG. As expected, survival outcomes are better in the VLRG versus no‐VLRG (5‐year PFS rate 93.4% vs. 92.1%; *P* = 0.37 and 5‐year OS rate 97.2% vs. 94.8%; *P* = 0.15), but no statistically significant difference was reached.

## Conclusions

Despite large studies have demonstrated a good diagnostic performance of SLN biopsy in cervical cancer patients, with a NPV ranging from 94.3% to 98.2%, one of the main concerns about its full adoption is the relatively low detection rate of the technique 11, 13, 20, 21, 22. As a matter of fact, looking at the official international literature, the frequency of a correct SLN identification really varies from one approach used to another and from a working team to another. In this context of very heterogeneous data, referring to the French and American studies that report the best results, the chance of optimal SLN identification is not superior to 76.5% 13, 15. These results significantly impact the overall sensitivity of the procedure, as a failure in SLN mapping in one or both the hemipelvis leaves the “negative” side completely uninvestigated. To improve sensitivity and NPV of the SLN technique up to 100%, and to reduce the false‐negative rate to 0%, a side‐specific nodal dissection has been introduced in any case of failed SLN mapping 15. In other words, if the best bilateral SLN detection rate reported in literature is about 75%, one quarter of women with early‐stage cervical cancer need to undergo at least unilateral LND to meet NCCN algorithm recommendations.

With the aim to increase the number of women to spare unnecessary pelvic LND, here we identified a subgroup of cervical cancer patient at very low risk (VLR) of LNM. We interestingly found that no patient, with negative MRI nodal assessment, with squamous and adenosquamous histotype and a tumor diameter less than 2 cm had LNM. This finding is consistent with previously reported data suggesting that squamous cervical cancer less than 2 cm in size has a LNM incidence ranging from 0% to 6% 23, 24. It is also worth noting that both histotype and tumor volume can be easily obtained preoperatively. In fact, MRI 25, 26 and transvaginal ultrasound 26, 27 show a very high diagnostic accuracy in measuring tumor volume. Moreover, tumor volume can also be evaluated intraoperatively at frozen section analysis.

Recently, consistent findings have been reported suggesting that SLN detection and consequently LND can be safely omitted in T1 apparently N0 breast cancer patients 28, 29.

Our data strongly support the hypothesis that a similar perspective can be tested in VLR early‐stage cervical cancer population, as identified in this study. The possibility to avoid even SLN detection in these patients may shorten and simplify cervical cancer surgery and histopathological examination, with a positive impact on patient benefit and costs. In such situation, primary tumor risk factors can be used for guiding adjuvant treatment. Moreover, leaving unaltered the lymphatic system might have another positive effect, thanks to the potential immunological surveillance of metastasis‐free LNs proximal to the tumor 30, 31. On the other hand although the clinical role of micrometastases is still far from being clarified, the risk of missing useful information avoiding ultrastaging of SLN biopsy cannot be denied.

**Figure 2:**
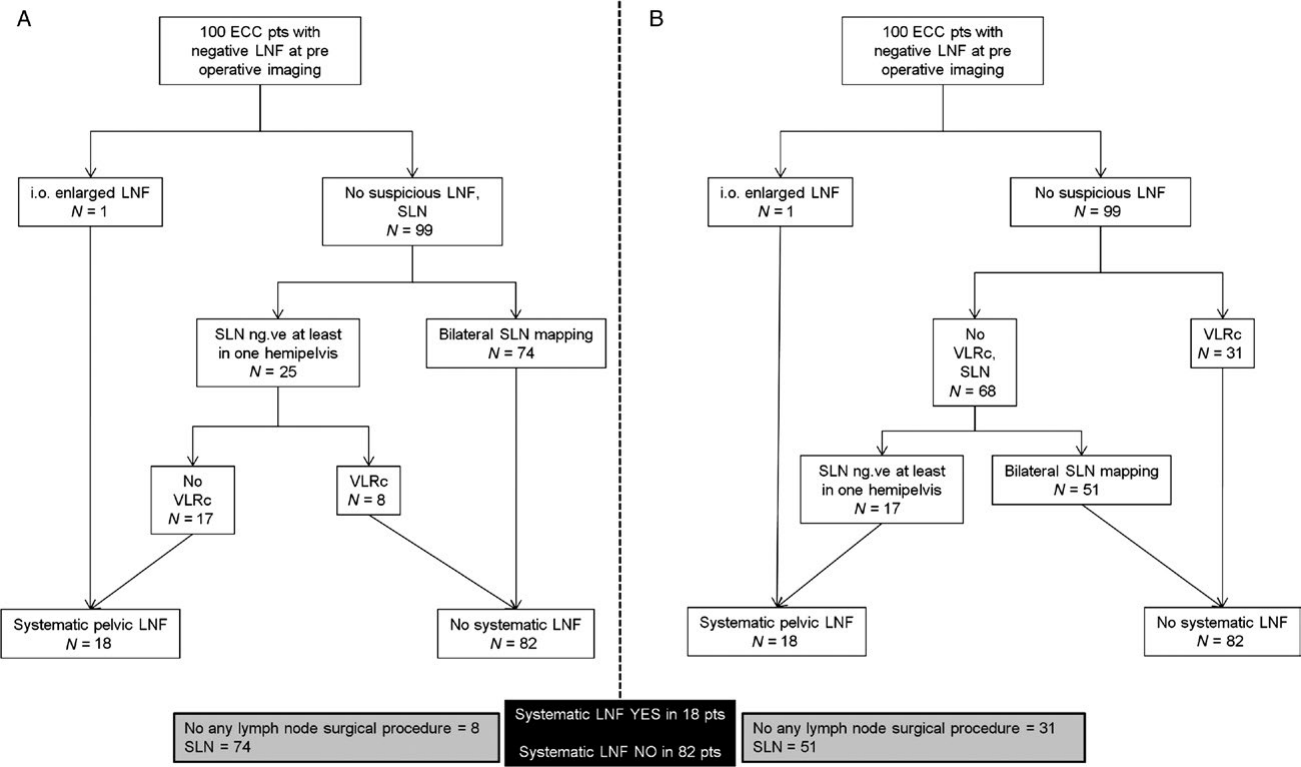
Distribution of lymphnodal assessment procedures in 100 patients with ECC and negative lymph nodal assessment at preoperative imaging. VLRG selection after SLN (A); VLRG selection before SLN (B). ECC, early cervical cancer; VLRG, very low‐risk group; SLN, sentinel lymph node.

Based on these considerations, we calculated two hypothetical clinical scenarios in 100 patients with early cervical cancer and negative lymph nodal assessment at preoperative imaging (Fig. 2). Figure 2A shows the case in which all women receive SLN staging. Here, eight patients with VLR factors among 25 with nonoptimal SLN mapping may safely avoid systematic LND. Figure 2B shows the case in which SLN is not performed in VLRG. Here, 31 VLR women do not receive any LN surgical procedure. In both cases, we obtain a significant reduction in the rate of LND performed, but the two approaches do differ in terms of lymph nodes (LFN) staging. In Figure 2A, the majority of patients (74/100) receive ultrastaging of bilateral SLNs, which also allows to detect lymph nodal micrometastases or Isolated tumor cells (ITC). Even if the benefit provided by the removal of such low‐volume lymph nodal disease is not already proven, the diagnosis of micrometastases is associated with a reduction in OS 32. This kind of approach does not interfere with the rate of ultrastaging procedures performed in a hypothetical group of 100 women, but provides benefit in the subset of patients in which optimal SLN detection failed, avoiding unnecessary LND (*n* = 8). On the other hand, in Figure 2B, only 51 of 100 women, instead of 74 receive ultrastaging, but 31 instead of eight safely avoid any lymph nodal surgical procedure. In this picture, we cannot exclude that some of the recurrences we registered in the VLRG could be predicted by nodal micrometastasis that SLN ultrastaging is able to detect. In other words, avoiding SLN detection may decrease the chances to correctly stratify risk categories. On the other side, the approach Figure 2B may decrease operative time and surgical costs, a relevant issue if we consider that cervical cancer is a social problem especially in developing countries with few resources and health facilities, including means to perform SLN technology (i.e., technetium or indocianine) and ultrastaging. Internal Hospital policy guidelines or clinical trials should define which one of these approaches is to be preferred.

In conclusion, our data suggest that in VLR early‐stage cervical cancer LND can be omitted when SLN is not detected. A prospective clinical trial is going to start in Italy comparing standard SLN protocol versus no LN assessment in this patient population.

## Conflict of Interest

None declared.
